# Hearing Aids Do Not Alter Cortical Entrainment to Speech at Audible Levels in Mild-to-Moderately Hearing-Impaired Subjects

**DOI:** 10.3389/fnhum.2020.00109

**Published:** 2020-04-03

**Authors:** Frederique J. Vanheusden, Mikolaj Kegler, Katie Ireland, Constantina Georga, David M. Simpson, Tobias Reichenbach, Steven L. Bell

**Affiliations:** ^1^Department of Engineering, School of Science and Technology, Nottingham Trent University, Nottingham, United Kingdom; ^2^Institute of Sound and Vibration Research, Faculty of Engineering and Physical Sciences, University of Southampton, Southampton, United Kingdom; ^3^Department of Bioengineering and Centre for Neurotechnology, Imperial College London, South Kensington Campus, London, United Kingdom; ^4^Audiology Department, Royal Berkshire NHS Foundation Trust, Reading, United Kingdom

**Keywords:** cortical entrainment, hearing impairment, hearing aid evaluation, speech detection, electroencephalography

## Abstract

**Background:**

Cortical entrainment to speech correlates with speech intelligibility and attention to a speech stream in noisy environments. However, there is a lack of data on whether cortical entrainment can help in evaluating hearing aid fittings for subjects with mild to moderate hearing loss. One particular problem that may arise is that hearing aids may alter the speech stimulus during (pre-)processing steps, which might alter cortical entrainment to the speech. Here, the effect of hearing aid processing on cortical entrainment to running speech in hearing impaired subjects was investigated.

**Methodology:**

Seventeen native English-speaking subjects with mild-to-moderate hearing loss participated in the study. Hearing function and hearing aid fitting were evaluated using standard clinical procedures. Participants then listened to a 25-min audiobook under aided and unaided conditions at 70 dBA sound pressure level (SPL) in quiet conditions. EEG data were collected using a 32-channel system. Cortical entrainment to speech was evaluated using decoders reconstructing the speech envelope from the EEG data. Null decoders, obtained from EEG and the time-reversed speech envelope, were used to assess the chance level reconstructions. Entrainment in the delta- (1–4 Hz) and theta- (4–8 Hz) band, as well as wideband (1–20 Hz) EEG data was investigated.

**Results:**

Significant cortical responses could be detected for all but one subject in all three frequency bands under both aided and unaided conditions. However, no significant differences could be found between the two conditions in the number of responses detected, nor in the strength of cortical entrainment. The results show that the relatively small change in speech input provided by the hearing aid was not sufficient to elicit a detectable change in cortical entrainment.

**Conclusion:**

For subjects with mild to moderate hearing loss, cortical entrainment to speech in quiet at an audible level is not affected by hearing aids. These results clear the pathway for exploring the potential to use cortical entrainment to running speech for evaluating hearing aid fitting at lower speech intensities (which could be inaudible when unaided), or using speech in noise conditions.

## Introduction

Accurate speech understanding is essential in day-to-day communication. There is currently much interest in gaining insight into the entrainment of neural activity in the auditory cortex to running speech stimuli (Zatorre et al., 2002; Schroeder and Lakatos, 2009; Giraud and Poeppel, 2012; Ding et al., 2017; Riecke et al., 2018). Cortical entrainment can be defined as the phase adjustment of neuronal oscillations in the auditory cortex to ensure high sensitivity to relevant (quasi-)rhythmic speech features (Lakatos et al., 2005; Giraud and Poeppel, 2012; Peelle et al., 2013; Alexandrou et al., 2018). These phase adjustments are considered to persist over time (Lakatos et al., 2005). Alternatively, cortical entrainment has been defined as the “observation of a constant phase of neural response to the same speech stimulus,” which avoids the need for an intrinsic relationship between the speech stimulus and neuronal oscillations (Alexandrou et al., 2018).

There is strong evidence that the neural activity in the auditory cortex entrains to low-frequency modulations in speech (in the delta-, theta-, and gamma-band, see e.g., Giraud and Poeppel, 2012; Ding et al., 2017). This idea stems from studies that showed high speech recognition of vowels and consonants after removing high-frequency spectral cues (Shannon et al., 1995). Furthermore, it has been shown that running speech shows a dominant frequency component in this slow modulation range resulting from rhythmic jaw movement associated with syllable structure in English (Peelle et al., 2013), especially around 4 Hz (Golumbic et al., 2012). When removing these low-frequency components, cortical envelope tracking is reduced, with the reduction in correlation consistent with a decrease in perceived speech intelligibility (Doelling et al., 2014). These results have led to the hypothesis that cortical entrainment to speech can be an objective measure for evaluating speech understanding and intelligibility and for an evaluation of hearing loss treatment strategies (Somers et al., 2018).

The exact electrophysiological mechanisms behind cortical entrainment to speech remain unclear. One electrophysiological model suggests that the auditory cortex segments (running) speech into discrete units based on temporal speech features, which allows cortical readout of syllabic and phonetic units (Giraud and Poeppel, 2012). Here, speech onsets are suggested to trigger a stronger coupling between theta-band (4–8 Hz) activity and gamma-band (25–40 Hz) activity cortical generators, with gamma-band activity controlling the excitability of neurons and theta-band activity tracking the temporal speech envelope. Cortical entrainment to these oscillations has also been suggested to be asymmetric, with theta-band activity more strongly represented in the right hemisphere and gamma-band activity more strongly represented in the left hemisphere (Morillon et al., 2012). Computer models of coupled theta-band/gamma-band coupling have further shown that theta-band activity can regulate a phoneme-level response based on gamma-band spiking activity (Hyafil et al., 2015). Studies have also found that theta-band phase-locking of the cortex to speech stimuli is an important mechanism for discriminating speech (Luo and Poeppel, 2007; Howard and Poeppel, 2012).

On the other hand, some studies have suggested that theta-band activity only reflects perceptual processing of speech, whereas delta-band (1–4 Hz) activity is involved with understanding speech (Molinaro and Lizarazu, 2018). This is in line with speech spectrum studies showing spectral peaks at sentence and word rates (0.5 Hz and 2.5 Hz, respectively) and showing that delta-band activity contains prosodic information which if removed reduces perceived speech intelligibility (Woodfield and Akeroyd, 2010; Edwards and Chang, 2013). One study comparing EEG responses to speech in noise at different frequencies with behavioral responses showed a decline in delta-band activity with reduced speech signal-to-noise ratio resembling the decline in subjectively rated speech intelligibility, whereas theta-band activity showed a linear decline (Ding and Simon, 2013). Another study showed that delta-band, low theta-band and high theta-band entrainment correspond to different features of the speech stream, indicating that both delta-band and theta-band entrainment might be necessary for optimal speech understanding (Cogan and Poeppel, 2011).

Several studies have focused on cortical responses to short speech like sounds using magneto- (MEG) or electro-encephalography (EEG) (Shahin et al., 2007; Ding and Simon, 2013; Millman et al., 2015; Mirkovic et al., 2015; Di Liberto et al., 2018a). Traditionally, these studies focused on evoked cortical responses to short stimuli such as words, consonants and vowel or speech-like tones (Friedman et al., 1975; Cone-Wesson and Wunderlich, 2003; Tremblay et al., 2003; Shahin et al., 2007; Van Dun et al., 2012). Often, these stimuli are short such that they can be repeated, which allows the signal-to-noise ratio of the cortical response to be enhanced through coherent averaging (Ahissar et al., 2001; Aiken and Picton, 2008), or through correlation between template and response EEG averages (Suppes et al., 1998). These studies have shown that cortical responses differ for different speech tokens (Cone-Wesson and Wunderlich, 2003; Tremblay et al., 2003) even at infancy (Van Dun et al., 2012). They have also been used to estimate auditory thresholds in adults (Lightfoot, 2016). Furthermore, it has been suggested that cortical evoked potentials can reflect speech-in-noise performance in children (Anderson et al., 2010).

The main issue with these approaches is that these short speech stimuli consist of individual, independent onsets and offsets to which the evoked response is measured. Running speech, however, is a continuous flow of onsets and offsets, which are dependent on the stimulus and adapt to the spectro-temporal structures of the stimulus. Running speech therefore gives the potential to track a collective of speech features (often referred to as cortical entrainment), rather than only onsets (evoked responses) (Ding and Simon, 2014). Another aspect of repeated short stimuli is that they do not reflect ecologically relevant stimuli that are encountered in everyday life (Alexandrou et al., 2018). In most cases, running speech is taken from audiobooks (Ding and Simon, 2013; O’Sullivan et al., 2014; Di Liberto et al., 2015; Mirkovic et al., 2015). Although still not exactly the same as naturally occurring every-day speech (dialogues), these stimuli are considered more relevant as they can be encountered in naturally occurring circumstances such as theater visits or news bulletins (Alexandrou et al., 2018).

Evaluation of cortical entrainment to running speech can be achieved through coherence analysis, with a focus on finding responses in the relevant frequency bands (Luo and Poeppel, 2007; Doelling et al., 2014). A technique that has gained much popularity for reconstructing running speech features from EEG (and MEG) signals is the temporal response function (TRF), which represents a linear mapping between features of the speech stimulus and the neural response (Lalor et al., 2006; O’Sullivan et al., 2014; Crosse et al., 2016a). TRF algorithms can be used either to predict EEG signals from stimulus features (forward model) or to reconstruct stimulus features from collected EEG signals (backward model) (Crosse et al., 2016a). Early studies used forward models to predict EEG responses to unseen stimuli based on a single stimulus feature (e.g., the speech envelope) (Lalor and Foxe, 2010). Recently, however, multivariate TRF models have been used to predict EEG responses in separate frequency bands based on speech spectrograms (Crosse et al., 2016a) and even phonetic features (Di Liberto et al., 2015). Similarly, although traditional backward models mostly attempted to only reconstruct a single feature from recorded EEG data (Ding and Simon, 2012, 2013, 2014; Mirkovic et al., 2015), multimodal algorithms have been developed that allow extracting information from both audio and visual features simultaneously (Crosse et al., 2016b). The TRF has shown the potential to identify an attended speaker in a cocktail party setting of many competing voices (Power et al., 2012; Horton et al., 2013; O’Sullivan et al., 2014), to predict speech-in-noise thresholds (Vanthornhout et al., 2018), to decode speech comprehension (Etard and Reichenbach, 2019), and has been used to investigate atypical speech processing in subjects suffering from dyslexia (Di Liberto et al., 2018b). TRF algorithms have also shown to have better response detection as compared to a cross-correlation analysis between EEG signals and speech stimuli (Crosse et al., 2016a).

Currently, interest is growing to apply TRF algorithms for evaluating hearing function and hearing aid fitting (Decruy et al., 2019). Better audibility, due to wearing a hearing aid, is expected to correlate with the level of cortical tracking of the speech envelope. Some studies have, however, shown that presenting vowel stimuli through hearing aids may affect cortical evoked responses, possibly due to the effect of hearing aid speech processing software on the speech spectrum (Easwar et al., 2012; Jenstad et al., 2012). The potential effect of hearing aid processing on cortical entrainment has, however, not yet been explored.

This study aimed to determine how cortical entrainment to the temporal envelope of running speech stimuli is affected by hearing aids in a cohort of mild-to-moderate hearing-impaired subjects when presenting the speech stimuli at an audible level. This was achieved by comparing the correlation between the original temporal speech envelope and the envelope reconstructed from EEG signals under aided and unaided conditions using a backward TRF algorithm. Mild to moderate hearing impaired subjects were chosen as they represent the largest group of users that are seen in typical hearing aid clinics (Suppes et al., 1998). If no effect of hearing aid processing would be observed, it would provide a first step toward objective audiological evaluation of hearing aid fitting using ecologically relevant stimuli, rather than clicks or tone stimuli (Billings, 2013). This might also facilitate the application of TRF algorithms in future real-time hearing aid speech processing, for example through providing input for optimization of hearing aid algorithms through cortical entrainment evaluations obtained from in-the-ear EEG systems (Mikkelsen et al., 2015).

## Materials And Methods

**FIGURE 1:**
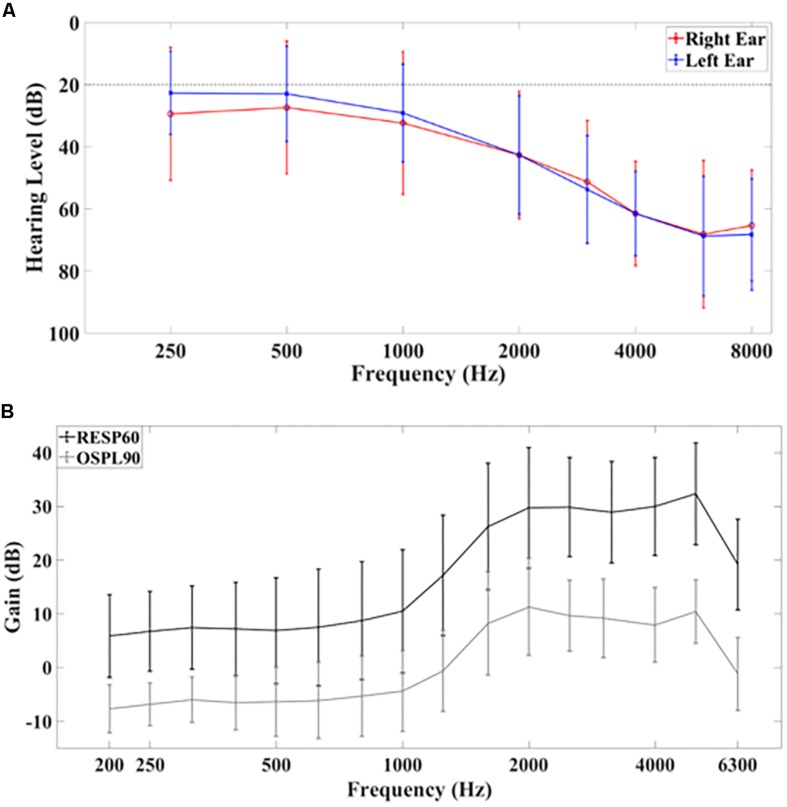
**(A)** Average hearing levels for the left and right ear based on pure tone audiometry. The dashed line at 20 dB indicates the threshold above which hearing is considered to be normal. **(B)** Hearing aid gain according to RESP60 (black) and OSPL90 (gray) tests. Error bars indicate ±1 standard deviation.

Seventeen native English-speaking subjects (11 males, 6 females, age 65 ± 5 years) with mild to moderate sensorineural and bilateral hearing impairment were recruited for this study (for full demographics, see Table 1). Hearing function was assessed through pure-tone audiometry (PTA). Figure 1A shows the average PTA hearing levels (in dB). Levels for the left ear at 250 Hz, 500 Hz, 1000 Hz, 2000 Hz, 3000 Hz, 4000 Hz, 6000 Hz, and 8000 Hz were: 23 dB ± 13 dB, 23 dB ± 15 dB, 29 dB ± 16dB, 43 dB ± 19 dB, 54 dB ± 17 dB, 61 dB ± 14 dB, 69 dB ± 19 dB, and 68 ± 18 dB, respectively (mean ± standard deviation). For the right ear, these values were: 29 dB ± 21 dB, 27 dB ± 21 dB, 32 dB ± 23 dB, 43 dB ± 21 dB, 51 dB ± 20 dB, 61 dB ± 17 dB, 68 dB ± 24 dB and 65 dB ± 18 dB, respectively.

**TABLE 1:** Subject demographics (HF, high frequency).

Patient ID	Gender (M/F)	Age (years)	Type of hearing loss	Symmetric hearing loss (yes/no)?	Hearing loss shape, best ear	Hearing loss shape, worst ear	Time since diagnosis (years)
01	F	65	Sensorineural	Yes	Flat	Flat	2
02	M	68	Sensorineural	No	Sloping HF	Flat	11
03	M	61	Sensorineural	Yes	Sloping HF	Sloping HF	14
04	F	52	Sensorineural	Yes	Ski slope	Ski slope	6
05	M	69	Sensorineural	No	Sloping HF	Sloping HF	7
06	M	69	Sensorineural	No	Sloping HF	Sloping HF	11
07	M	70	Sensorineural	Yes	Sloping HF	Sloping HF	3
08	F	70	Sensorineural	No	Sloping HF	Sloping HF	9
09	M	68	Sensorineural	No	Sloping HF	Sloping HF	6
10	M	66	Sensorineural	No	Sloping HF	Sloping HF	9
11	F	57	Sensorineural	Yes	Sloping HF	Sloping HF	13
12	M	68	Sensorineural	Yes	Sloping HF	Sloping HF	11
13	M	64	Sensorineural	No	Sloping HF	Sloping HF	2
14	F	70	Sensorineural	Yes	Flat	Flat	18
15	F	57	Sensorineural	No	Sloping HF	Sloping HF	2
16	M	65	Sensorineural	Yes	Sloping HF	Sloping HF	11
17	M	67	Sensorineural	Yes	Sloping HF	Sloping HF	9

All subjects had hearing aids fitted binaurally based on NAL-NL2 guidelines with real-ear measurements (see Table 2 for hearing aid features) (Aazh and Moore, 2007; Keidser et al., 2011). The average use of hearing aids over all subjects was 84 ± 48 months (mean ± standard deviation, range 4–191 months). The average real ear hearing aid gain for ISTS noise at 60 dB SPL input (RESP60, black) and the output sound pressure level with input of 90 dB SPL and gain full on (OSPL90, gray) measurements are shown in Figure 1B. Speech understanding was further evaluated by asking the participants to repeat a set of randomized Bamford-Kowal-Bench (BKB) sentences (Bench et al., 1979) presented at 65 dBA SPL under aided and unaided conditions. The sentence list was different for both conditions. All participants gave informed consent for the study. The study was approved by the local National Health Service (NHS) ethics committee.

**TABLE 2:** Overview of hearing aid settings (SS, soft switching).

Patient ID	Hearing aid type	Time using hearing aid (months)	Hearing aid program settings	Noise reduction	Frequency lowering
01	GN resound UP967 open fit	51.0	Basic, party, T+M	No	No
02	GN resound UP988 R and UP977 L plus closed EMs	83.0	Basic+SS, party, T+M	No	No
03	GN resound UP967 open fit	141.0	Directional, restaurant	No	No
04	GN resound UP967 open fit	43.0	Basic	No	No
05	GN resound UP977 open fit	49.0	Basic+SS, restaurant, T	No	No
06	GN resound UP967 open fit	93.0	Basic+SS, party, music, T+M	No	No
07	GN resound UP967 open fit	4.0	Basic, restaurant, T	No	No
08	GN resound UP977 and vented EMs	85.0	Basic, party	No	No
09	GN resound UP967 open fit	43.0	Directional, restaurant, T	No	No
10	GN resound UP977 open fit	81.0	Basic+SS, party	No	No
11	GN resound UP967 open fit	126.0	Directional	No	No
12	GN resound UP967 open fit	104.0	Basic+SS, restaurant, T	No	No
13	GN resound UP967 open fit	5.0	Basic, party, T+M	No	No
14	GN resound UP977 plus EMs	191.0	Basic, party, T+M	No	No
15	GN resound UP967 open fit	125.0	Basic+SS, party	No	No
16	GN resound UP967 open fit	109.0	Basic, party	No	No
17	GN resound UP967 open fit	89.0	Basic, party, T	No	No

Subjects were asked to listen to eight running speech segments of about 3 min each under aided and unaided conditions (total stimulus length of about 25 min). The stimulus was taken from a freely available audiobook^1^ and presented by a female speaker. Speech was sampled at 44,100 Hz and low-pass filtered at 3,000 Hz using 120th order finite impulse response (FIR) filter before presentation. Conditions were randomized amongst subjects. Segments were presented at 70 dBA equivalent sound pressure level (LeqA SPL) through a loudspeaker positioned 1.2 m directly in front of the subject. After each segment, participants were asked multiple-choice questions about the segments’ contents to determine if they paid attention to and understood the speech. Simultaneously, EEG data were collected using a 32-channel EEG system (BioSemi, Netherlands, sampling rate 2048 Hz) with two additional electrodes positioned at either mastoid. The electrodes were positioned according to the standard 10–20 system and referenced to the average EEG signal over all electrodes.

Objective assessment of speech understanding was based on measuring the entrainment of slow neural oscillations to speech, by correlating the actual stimulus speech envelope with that reconstructed from the EEG data using a linear model.

The recorded EEG was bandpass filtered (FIR filter, Hamming window, one-pass forward and compensated for delay) according to distinct frequency bands of slow neural oscillations. In particular the corner frequencies of the applied zero-phase filters were 1–4 Hz (transitions bandwidth: 1 Hz (low), 2 Hz (high), order 6759), 4–8 Hz (transitions bandwidths: 2 Hz (low), 2 Hz (high), order 3379), and 1–20 Hz (transitions bandwidths: 1 Hz (low), 5 Hz (high), order 6759), corresponding to the delta-, theta-, and broad-band EEG activity, respectively. The resulting signal was furthermore down-sampled to 64 Hz. All the above specified pre-processing steps were performed using functions from the MNE python package (Gramfort et al., 2013, 2014).

To extract the temporal speech envelope from the stimulus, an absolute value of its analytic signal was computed. Specifically, the analytic signal was a complex signal composed of the original stimulus as a real part and its Hilbert transform as an imaginary part. The stimulus’ temporal speech envelope obtained this way was subsequently filtered and down-sampled in the same way as the EEG recordings.

To reconstruct the stimulus’ temporal speech envelope from the EEG data, a spatiotemporal model was established. Specifically, at each time instance *t*_*n*_, the temporal speech envelope *y*(*t*_*n*_) was estimated as a linear combination of neural recording *x*_*j*_(*t*_*n*_ + τ_*k*_) at a delay τ_*k*_:

$$\hat{y}\,\left(t_{n}\right)=\sum_{j=1}^{N}\sum_{k=1}^{T}\left[\beta_{j,k}\,x_{j}\,\left(t_{n}+\tau_{k}\right)\right]$$

The index *j* refers to the recording channel, τ_*k*_ to the delay of the EEG with respect to stimulus ranging from −100 ms to 400 ms and β_*j,k*_ is a set of the decoder’s weights. For each subject, to obtain the model coefficients, a regularized ridge regression was applied: β = *X^t^X* + λ*I*)^−1^*X^t^y*, where *X* is the design matrix, *X^t^* is the transpose of *X*, λ is a regularization parameter and *I* is an identity matrix. *NT* columns of the design matrix correspond to recording channels at different latencies *x*_*j*_(*t*_*n*_ + τ_*k*_) and each row represents a different time *t*_*n*_.

To evaluate the reconstruction performance of the decoder, for each participant, a five-fold cross-validation procedure was applied. In each of five iterations, 80% of the data (∼20 min) was used to estimate the model and the remaining 20% (∼5 min) was employed to reconstruct the temporal speech envelope from the EEG (ŷ=*X*β). The reconstructed envelope and the actual (*y*) were subsequently divided into ten-seconds long parts (∼30 segments per fold of data) and the Pearson’s correlation coefficient between the two was computed for each of the obtained segments. For each subject, 50 different regularization parameters with values ranging from 10^–15^ to 10^15^ were tested to optimize the decoder. The optimal regularization parameter was the one that yielded the largest correlation coefficient averaged across all the testing folds and segments. For the optimal regularization parameter, correlation coefficients obtained from all the testing segments, across all the five folds were then pooled together to form a single distribution. Mean and standard deviation of this distribution reflected the envelope reconstruction performance of the optimized decoder.

To assess the empirical chance level reconstruction performance, the same procedure, including the same cross-validation and the optimization of the regularization parameter, was applied but the temporal speech envelope was reversed in time. The obtained correlation coefficients from short testing segments were similarly pooled together across all the five folds to form a null distribution. The chance-level correlations were subsequently compared to those obtained from the forward speech model, using the same methodology, via a Wilcoxon signed-rank test.

As Pearson’s correlation coefficients, used here to assess the temporal speech envelop reconstruction, were non-normally distributed, non-parametric tests were used during the study. EEG correlations and behavioral results under aided and unaided conditions were compared using a Wilcoxon signed-rank test. A Kruskal–Wallis test was used for comparing differences in variances. Linear correlations between cortical entrainment correlations and behavioral data were fitted using a bisquare robust regression algorithm. As two subjects could not complete the BKB sentence test due to experiments overrunning, their EEG data were excluded for this part of the study. Significance was assessed after adjusting for multiple comparisons based on expected false-discovery rates according to the Benjamini–Yekutieli algorithm (Benjamini and Yekutieli, 2001). Note that this adjustment allows for *p*-values to be higher than 1.

## Results

**FIGURE 2:**
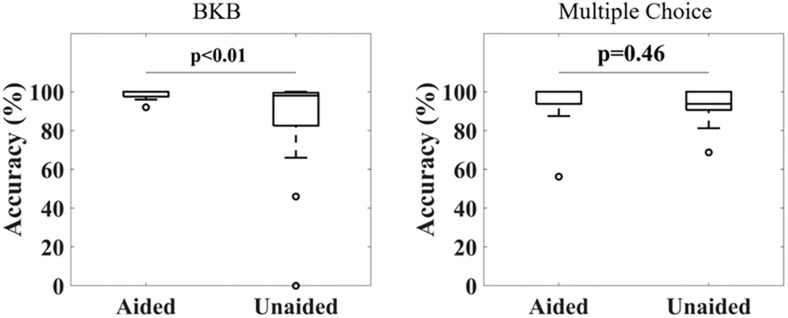
Distribution of correct response ratios under aided and unaided conditions for a BKB sentence list **(Left)** and multiple choice questions related to the audiobook **(Right)**. A significant difference in accuracy was observed for the BKB sentence lists under aided compared to unaided conditions, but not for the multiple choice questions (Wilcoxon signed-rank test, α = 0.05).

Figure 2 shows the accuracy in repeating the BKB sentences (left) and answering the multiple-choice questions (right) for all subjects under aided and unaided conditions. For the BKB sentences, 16 out of 17 subjects achieved a score above 95% under aided conditions, with the other subject scoring 92% (mean ± standard deviation: 98% ± 2%). The scores for unaided conditions were distributed over a larger range, with four subjects scoring less than 90% and another three subjects scoring below 95% (mean ± standard deviation: 84% ± 28%). Applying a Wilcoxon signed-rank test showed a significant difference in the distribution means of both conditions (*p* < 0.001). A significant difference could still be observed after removing data from two outliers scoring <50% in the unaided condition (*p* = 0.04). Similarly, the Kruskal–Wallis test showed a significant difference in variance between both conditions (*p* = 0.032). For the multiple-choice questions, high accuracy was obtained for both aided (mean ± standard deviation: 95% ± 10%) and unaided (mean ± standard deviation: 92% ± 11%) conditions. No significant difference between the distributions was found (Wilcoxon signed-rank test).

**FIGURE 3:**
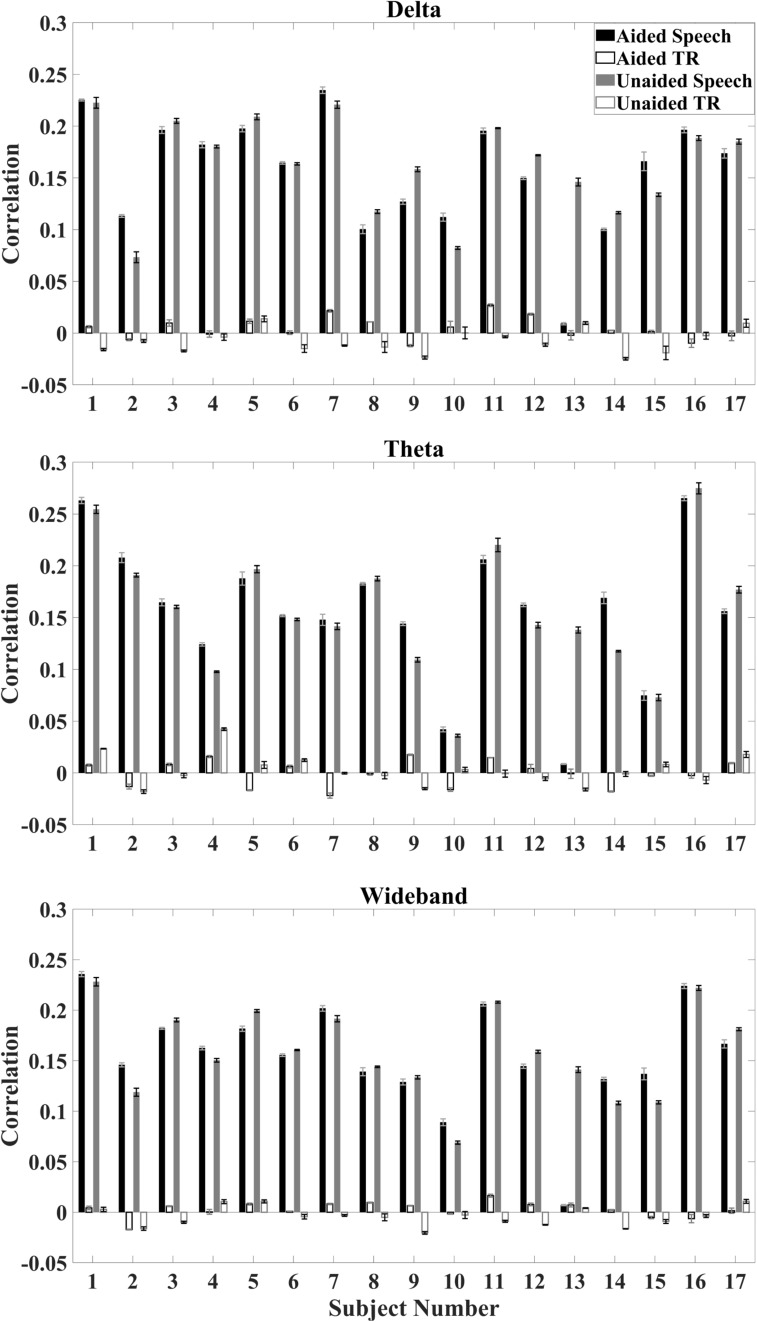
Average correlation for individual subjects under aided and unaided conditions for speech envelopes reconstructed from EEG signals to the aligned speech and time-reversed (TR) speech envelope. Error bars indicate standard deviations. **Top:** delta activity; **Middle:** theta activity; **Bottom:** wideband activity.

Correlations between the reconstructed temporal envelope based on the decoder algorithm and the time-aligned as well as time-reversed speech envelope were evaluated on the individual subject and the population level. On an individual level, the median correlation between reconstructed envelopes and the aligned speech envelope is higher than the median correlation between the reconstructed envelopes and time-reversed speech envelope for all subjects (Figure 3).

**FIGURE 4:**
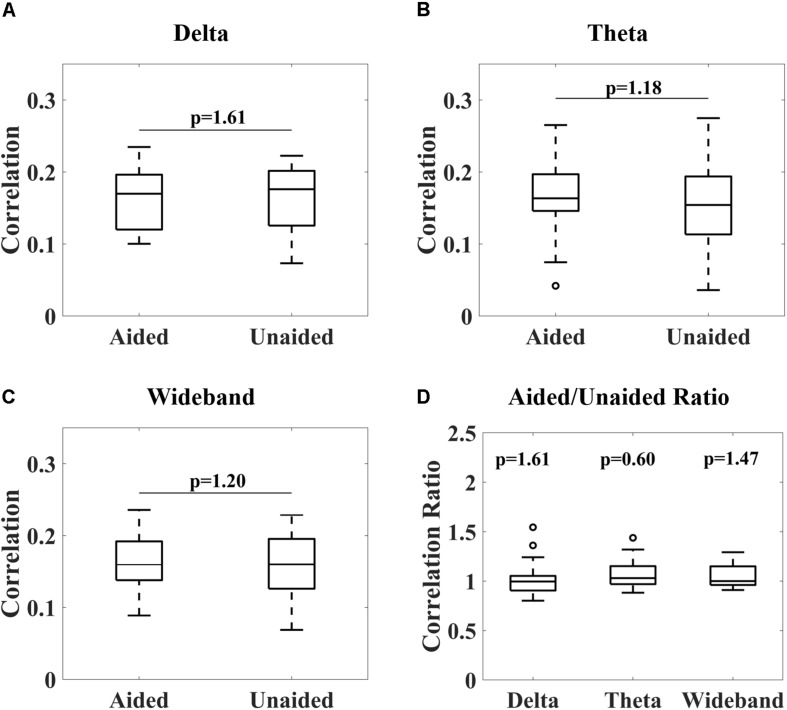
Correlations between reconstructed and real audiobook speech envelopes for delta-band (1–4 Hz, **A**), theta-band (4–8 Hz, **B**) and wideband (1–20 Hz, **C**) activity under aided and unaided conditions. No significant differences in distribution could be observed (Wilcoxon signed-rank test, *p*-values adjusted for multiple comparisons according to Benjamini–Yekutieli algorithm). A Wilcoxon signed-rank test comparing aided/unaided ratios further showed these ratios were not significantly different from 1 **(D)**.

Figure 4 shows the overall distribution of correlations between the reconstructed and speech envelope for the different EEG bands under aided and unaided conditions for the remaining subjects. Generally, results show that hearing aids did not significantly alter cortical entrainment to the speech envelope. For all EEG bands, correlations varied between 0.07 and 0.24 for wideband and delta-band activity and 0.04 and 0.27 for theta-band activity over all subjects except subject 13. After checking the power spectral density function, it was observed that a technical issue occurred while collecting subject 13’s data, which were therefore removed from further analysis.

**FIGURE 5:**
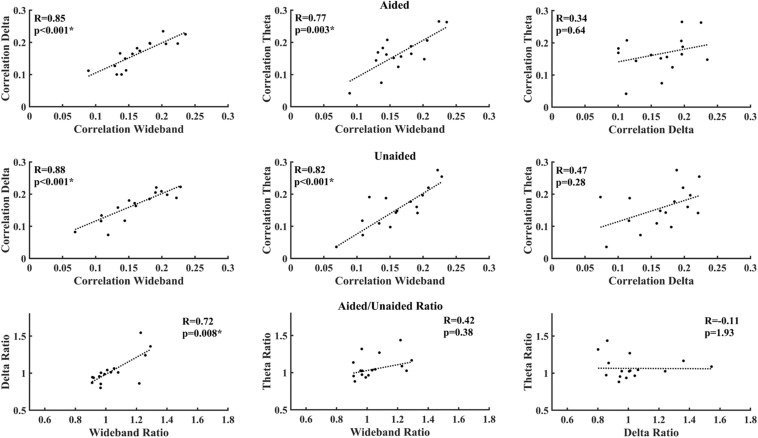
Correlation between average decoder correlation values under aided **(Top)** and unaided conditions **(Middle)**, as well as between aided/unaided ratios **(Bottom)**. Asterisks indicate significant correlations after correcting for multiple comparisons (Benjamini–Yekutieli adjusted *p*-values).

Figure 5 shows the correlations between different EEG bands under aided and unaided conditions. Both delta-band and theta-band activity show a strong and significant correlation after correcting for multiple comparisons, with the wideband activity under aided and unaided conditions. However, the correlation between delta-band and theta-band activity is lower and not significant.

**FIGURE 6:**
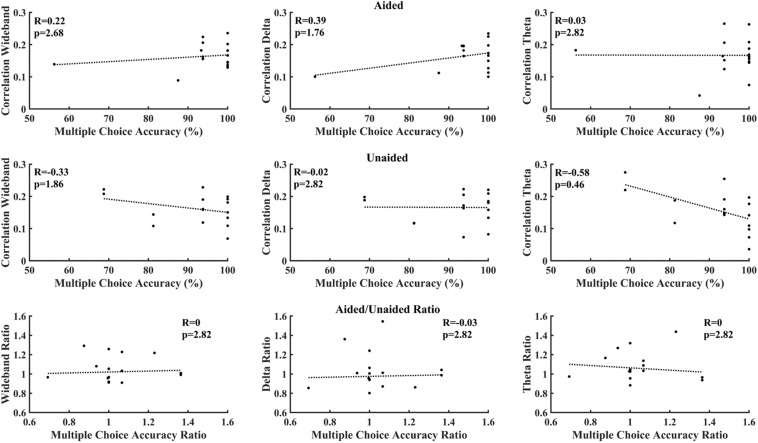
Correlation between multiple choice accuracy and decoder reconstruction accuracy. **Top:** aided; **Middle:** unaided; **Bottom:** ratio (aided/unaided). No significant correlations could be observed between EEG activity and multiple-choice accuracy after correction for multiple comparisons (Benjamini–Yekutieli adjusted *p*-values).

**FIGURE 7:**
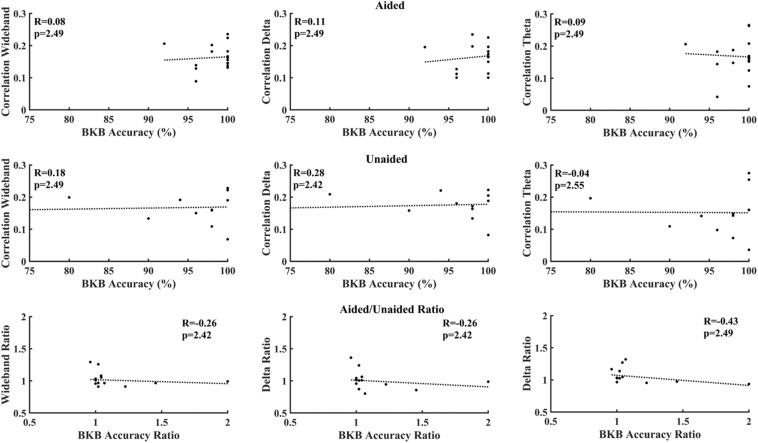
Correlation between BKB test accuracy and decoder reconstruction accuracy. **Top:** aided; **Middle:** unaided; **Bottom:** ratio (aided/unaided). No significant correlations could be observed between EEG activity and multiple-choice accuracy after correction for multiple comparisons (Benjamini–Yekutieli adjusted *p*-values).

Figure 6 correlates cortical entrainment with the multiple-choice scores. No significant correlations were found between the EEG activity and multiple-choice scores after correcting for multiple comparisons (Benjamini–Yekutieli adjusted *p*-value). Changes in cortical entrainment did also not correlate with change in multiple-choice scores when taking the difference in correlation in cortical entrainment and multiple-choice scores between aided and unaided conditions. Similarly, no significant correlations could be found between behavioral scores obtained from BKB sentences and neural entrainment (Figure 7).

## Discussion

Evaluation of low-frequency cortical entrainment to speech stimuli has been suggested as a potential indicator of speech understanding and intelligibility (Cogan and Poeppel, 2011; Ding and Simon, 2013; Di Liberto et al., 2015; Vanthornhout et al., 2018), and therefore evaluation of hearing function and hearing aid fitting. However, the effect of hearing aid speech processing software on this entrainment has not yet been investigated (Easwar et al., 2012; Jenstad et al., 2012). This study investigated if cortical entrainment to temporal speech envelopes is affected when presenting the speech stimuli at an audible level under aided against un-aided conditions in a cohort of mild-to-moderate hearing-impaired subjects. As the speech was audible for all our subjects, differences in cortical entrainment observed would be caused by the application of hearing aids. Speech stimuli used in this study were taken from an audiobook. Although not equal to and less frequently occurring in everyday life compared to natural conversations, this type of stimulus is considered more ecologically relevant compared to repeating sentences (Alexandrou et al., 2018).

Behavioral results (Figure 2) showed that subjects were able to hear the speech, and that under aided conditions, subjects performed significantly better in reproducing BKB sentences. One subject had a lower score of 92% accuracy under aided conditions, which could not be explained through demographic or hearing aid characteristics nor the duration of hearing aid use. However, we did not test our subjects for cognitive impairment, which even in mild conditions may affect performance in speech tests and has been shown to affect cortical and brainstem responses to sound stimuli (Moore et al., 2014; Bidelman et al., 2017). For the multiple-choice questions, higher variability in performance could be observed compared to BKB sentences, which again could not be directly explained through demographics or hearing aid features. Apart from the suggested effect of cognitive function, this might have been due to increased fatigue as it has been shown that listening attentively to long-duration speech stimuli requires more effort for hearing impaired subjects (Nachtegaal et al., 2009; Ayasse et al., 2017).

Based on decoder analysis for individual subjects, no specific trend between aided and unaided conditions could be observed (Figure 3). Some subjects show a higher correlation under aided conditions, whereas others show a higher correlation under unaided conditions. One subject (subject 13) showed a specifically low correlation for the time-aligned speech under aided conditions for all EEG bands.

This gave cause to analyze if results were confounded by which condition was used first in presenting the speech stimulus. Since a sign test showed that the median difference in correlation between the first and second condition played to each subject was not significantly different from 0, it could be determined that there was no correlation between the strength of the correlation and which condition was played first. This indicates that, for the remaining subjects, differences in correlation were not due to a lack of attention during the repeat of the stimulus.

From Figure 4, it can be observed that no significant differences in cortical entrainment occurred for either delta-band, theta-band or wideband activity (Wilcoxon signed-rank test, Benjamini–Yekutieli adjusted *p*-value). Unaided against aided correlation ratios were also tested for each frequency band to determine if trends in cortical entrainment could be observed (Figure 4D). However, this analysis also failed to show any significant trend. Results of this study are similar to correlations found in previous studies, which mostly used study populations consisting of younger test subjects (Ding and Simon, 2012; Crosse et al., 2016b; Vanthornhout et al., 2018). These results are probably caused by the speech stimuli being presented at a comparatively high intensity, well audible for the participants, even without their hearing aid. The good audibility already presents in the unaided condition, corroborated by the good unaided speech comprehension (Figure 2, right), lead to a significant neural response to the unaided speech envelope that presumably was not further increased by the hearing aid. The rationale for using quite a high speech level of 70 dB (A) was to ensure good entrainment to speech was possible for subjects even under unaided conditions (Etard and Reichenbach, 2019), and therefore establish that hearing aids do not significantly alter this entrainment under fully audible conditions. Due to long test durations, we could not explore the effect of lower intensity stimuli in this study. Evaluations of cortical entrainment under lower intensity stimuli conditions will be an important area of future research to determine if changes in cortical entrainment can be observed when hearing impaired subjects are listening to stimuli only audible under aided conditions, and therefore if cortical entrainment can find applications in hearing aid fitting evaluation.

Although hearing aid processing may alter the temporal speech envelope, our data shows that such alterations do not significantly alter the cortical entrainment. It could be that the decoder technique is robust to small changes in the speech envelope, which would be in agreement with a previous study that showed that different types of computing amplitude modulation of speech - using the Hilbert envelope or a more involved model of the auditory periphery with an auditory filter bank and non-linear compression - did not greatly affect the neural entrainment as measured from scalp EEG (Biesmans et al., 2016). This study, therefore, shows that the application of hearing aids does not significantly affect cortical entrainment of speech in quiet at a sound intensity above the hearing threshold. This provides some reassurance that cortical entrainment to speech can be evaluated in future studies to determine its potential in improving hearing aid fitting strategies for speech presented in noise or at an intensity below threshold for mild to moderately impaired hearing subjects (where the hearing aid should then make the speech audible). Recent studies have already shown that cortical entrainment might have potential in assessing hearing function in severely hearing-impaired subjects who have received a cochlear implant (Somers et al., 2018).

Correlations between activities of individual EEG bands are shown in Figure 5. Although both delta-band and theta-band entrainment showed a strong and significant correlation with the wideband activity as expected, the correlation between delta-band and theta-band activity was not significant. This reduced correlation possibly indicates that delta-band and theta-band activity entrain to different features of speech, as suggested in previous work (Giraud and Poeppel, 2012; Ding and Simon, 2013, 2014). For aided-unaided ratios, the only strong and significant correlation could be observed between the wideband and delta-band ratio, suggesting that wideband activity might be mostly driven by delta-activity.

Figures 6, 7 evaluate the correlation between cortical entrainment and behavioral results (multiple choice questions and BKB sentence analysis) for all EEG frequency bands of interest. No significant correlation between cortical entrainment and behavioral results could be observed, possibly because the fluctuations in cortical entrainment were larger than those in behavioral scores and the low number of participants in the current study. Another study evaluating cortical entrainment to speech in noise in normal-hearing subjects did find a correlation between strength of entrainment and behavioral responses (Vanthornhout et al., 2018). Further studies on larger hearing-impaired cohorts will be required to evaluate the strength of the correlation between behavioral responses to speech and cortical entrainment under aided and unaided conditions with speech in quiet and noise to determine the applicability of cortical entrainment analysis on hearing aid fitting evaluation.

Another interesting aspect was that trends in behavioral responses to speech (multiple-choice questions) differed to those of BKB sentences. Correct response ratios for BKB sentences under unaided conditions were significantly lower than under aided conditions, whereas no difference could be found for multiple-choice questions (Figure 2). Apart from a difference in intensity, a possible reason for this is that it might be easier to derive the correct answer due to having a context in running speech. Answers to the multiple-choice questions were often repeated during the audiobook or could be derived from the storyline. This repetition can lead to mind wandering and loss of attention, yet studies have shown that this would only affect performance in case strong detachment from the task occurs (Mooneyham and Schooler, 2016). Through observation, we were able to ensure participants were never fully losing attention to the speech stimulus. With audiobooks speech stimuli generally being clear and at a lower pace than natural conversations, repetition might have improved recalling answers to the multiple choice questions under unaided conditions, as studies have shown reduced speech rate can decrease cognitive load (Donahue et al., 2017; Millman and Mattys, 2017). BKB sentences on the other hand are independent from one another and not repeated, preventing subjects deriving correct answers by using context or recall from memory.

## Conclusion

This paper investigated if cortical entrainment to running speech is affected by hearing aid processing in a cohort of mild-to-moderately impaired subjects. Speech was presented at audible levels in aided and unaided conditions. Results show that measurement of entrainment to the temporal speech envelope is reliable with and without hearing aids. At these levels hearing aids do not significantly alter cortical entrainment to the speech envelope acquired before hearing aid processing, however. As speech was presented at an audible level, behavioral data indicated high understanding of the unaided speech stimulus. No significant correlation between cortical entrainment and behavioral data could be found. Future studies measuring cortical entrainment to speech in more challenging conditions, for example presented at or below subject-specific hearing levels or in a noisy environment could further clarify the potential of cortical responses to optimize hearing aid fitting evaluation.

## Data Availability Statement

All data supporting this study are openly available from the University of Southampton repository at https://doi.org/10.5258/SOTON/D1140.

## Ethics Statement

The studies involving human participants were reviewed and approved by local National Health Service (NHS) ethics committee. The patients/participants provided their written informed consent to participate in this study.

## Author Contributions

FV, KI, DS, TR, and SB contributed to the conception and design of the study. FV, KI, and CG organized the database and data collection. FV and MK performed data analysis and statistical analysis. FV wrote the first draft of the manuscript and MK wrote sections of the manuscript. All authors contributed to manuscript revision, read and approved the submitted version.

## Conflict of Interest

The authors declare that the research was conducted in the absence of any commercial or financial relationships that could be construed as a potential conflict of interest.

## Footnotes

1 https://librivox.org/the-children-of-odin-by-padraic-colum/ (Chapters 2 and 5)
